# Dynamic prediction of hospital admission with medical claim data

**DOI:** 10.1186/s12911-019-0734-y

**Published:** 2019-01-31

**Authors:** Tianzhong Yang, Yang Yang, Yugang Jia, Xiao Li

**Affiliations:** 1 Philips Research North America, Cambridge, MA 02141 USA; 2 Department of Biostatistics and Data Science, The University of Texas Health Science Center at Houston, Houston, 77030 TX USA

**Keywords:** Claim data, Survival analysis, Dynamic prediction, Random survival forest, Sliding window, Congestive heart failure, Hospitalization

## Abstract

**Background:**

Congestive heart failure is one of the most common reasons those aged 65 and over are hospitalized in the United States, which has caused a considerable economic burden. The precise prediction of hospitalization caused by congestive heart failure in the near future could prevent possible hospitalization, optimize the medical resources, and better meet the healthcare needs of patients.

**Methods:**

To fully utilize the monthly-updated claim feed data released by The Centers for Medicare and Medicaid Services (CMS), we present a dynamic random survival forest model adapted for periodically updated data to predict the risk of adverse events. We apply our model to dynamically predict the risk of hospital admission among patients with congestive heart failure identified using the Accountable Care Organization Operational System Claim and Claim Line Feed data from Feb 2014 to Sep 2015. We benchmark the proposed model with two commonly used models in medical application literature: the cox proportional model and logistic regression model with L-1 norm penalty.

**Results:**

Results show that our model has high Area-Under-the-ROC-Curve across time points and C-statistics. In addition to the high performance, it provides measures of variable importance and individual-level instant risk.

**Conclusion:**

We present an efficient model adapted for periodically updated data such as the monthly updated claim feed data released by CMS to predict the risk of hospitalization. In addition to processing big-volume periodically updated stream-like data, our model can capture event onset information and time-to-event information, incorporate time-varying features, provide insights of variable importance and have good prediction power. To the best of our knowledge, it is the first work combining sliding window technique with the random survival forest model. The model achieves remarkable performance and could be easily deployed to monitor patients in real time.

**Electronic supplementary material:**

The online version of this article (10.1186/s12911-019-0734-y) contains supplementary material, which is available to authorized users.

## Background

There is a great need for health care service providers, such as Accountable Care Organization (ACO), to closely monitor the health conditions of their registered beneficiaries, predict their future adverse events, and then take prompt actions to alleviate or prevent such events [1]. The Centers for Medicare and Medicaid Services (CMS) has established the ACO model where groups of health care providers could unite to form their own ACO for Medicare beneficiaries. CMS releases the Claim and Claim Line Feed (CCLF) files monthly which contain up-to-date beneficiary-level claim feed data. The traditional batch-mode models used for static data may not achieve the best prediction performance. Currently, there are few prediction models built on such continuously updated medical files. To fully utilize such data, we developed a novel model called the dynamic random survival forests (DRSF) model, which combines the advantage of data stream models and random survival forest (RSF) model. The model updates adaptively with time-varying features that balance between the comprehensiveness and the timeliness of historical information. It also predicts the risk of future adverse events in a day unit for every beneficiary with few model assumptions. We demonstrate that our DRSF model is effective and powerful to make risk prediction and stratification for periodically updated medical data and can be deployed to monitor patients in real time.

In this paper, we focus on hospitalization caused by Congestive Heart Failure (CHF) as our future adverse events. CHF is a serious medical condition in which the heart cannot pump enough blood to meet the body needs. It is one of the most common reasons those aged 65 and over are hospitalized in the United States [2, 3]. CHF has created considerable economic burdens in the elderly and has been estimated to account for approximately 1 to 2% of the annual total costs to a health care system [4, 5]. On the other hand, CHF is a highly preventable condition. Taking appropriate medication, proper management of hypertension, and routine checkups can help ensure the prevention of heart failure. Therefore, a precise prediction of hospitalization caused by CHF in the near future could advise ACOs to avoid possible hospitalization, optimize their medical resources and better meet the healthcare needs of beneficiaries.

The CCLF claims data are provided by CMS on a monthly basis to Medicare ACOs. The CCLF files released by CMS contain rich beneficiary-level information covering the health conditions and all visiting episodes of beneficiaries. Besides, the files are continuously released to the participating ACOs in a unified format, so it is cost-effective to extract the beneficiary’s information from these files. However, it raises statistical challenges regarding efficiently utilizing such high-volume and high-velocity data. Our proposed dynamic survival predictive model, the DRSF model, is able to accommodate those requirements. To our knowledge, this is the first work that combines data stream model and RSF model with a real application to predict adverse events using claim data. Shaker and Wang proposed to combine a Cox-like proportional model and data stream [6, 7]. However, their work focused on examining the influence of covariates changing over time rather than predicting adverse events. The following sections discuss the details of our method and the application of our model in predicting hospitalization caused by CHF. We apply our method to the CCLF claims data from Feb 2014 to Sep 2015 for predicting hospitalization caused by CHF. We compare our model with traditional batch-mode models that ignore temporality. In addition, we explore the validity of our assumption whether the recent past is more informative for prediction purposes than the older.

## Methods

### Study population

Our study cohort consists of those with previous CHF diagnoses who are at high risk of future hospitalization due to CHF. Alexian Brother ACO, locating in the suburbs of Chicago, IL, serves over 60,000 Medicare beneficiaries. We collected all the data necessary for this study within Alexian Brother ACO from the CCLF files. The high-risk beneficiaries were identified as patients who had CHF comorbidity in the past one year and the CHF comorbidity was recognized by the historical International Classification of Diseases, Ninth Revision, Clinical Modification (ICD-9-CM) diagnostic codes in the CCLF files. To subset and retain members with CHF diagnosis, we used Quan’s updated Elixhauser’s Comorbidity definition to define the CHF cohort with the corresponding ICD-9-CM codes, i.e., 398.91, 402.01, 402.11, 402.91, 404.01, 404.03, 404.11, 404.13, 404.91, 404.93, 428, 428.0, 428.1, and 428.9 as combined from Braunstein et al. [8] and Zolfaghar et al. [9]. To identify the beneficiaries with high risk based on the most recent data, we collected CHF cohort at different index dates, i.e., in a dynamic manner. For example, if index date is set at Feb 1, 2014, we use claim data between Feb 1, 2013 and Jan 31, 2014 (12 months) to obtain the evidence of CHF comorbidity and establish the CHF cohort. In this way, we collected 14 CHF cohorts corresponding to 14 index dates, with the sample size ranging from a minimal of 4114 to a maximal of 5231 with a mean of 4869 patients. The index dates covered from Feb 1, 2014 to Apr 1, 2015. A selected feature descriptive summary (of patient cohort with an index date of Feb 1, 2014) is presented in Table 1. Before collecting any data, we signed the Data Use Agreement Addendum for Data Acquired from the CMS and obtained the approval.

**Table 1:** Descriptive Statistics of Baseline Features of CHF cohort with an index date of Feb 1, 2014

	Without CHF hospitalization	Encounter CHF hospitalization till the end of study^a^	Distribution Significance (*p*-value)^b^
# of members (% in total)	4910 (94.7%)	273 (5.3%)	
Demographic Status			
Gender (% of Male in total)	43.50%	47.60%	0.199
Ethnicity (# of members in 0/1/2/3/4/5/6 categories)^c^	22/4470/261/51/65/38/3	0/245/20/4/2/2/0	0.62
Age (mean age)	80.8	82.6	0.004
Beneficiary Medicare Status Code (# of members in 10/11/20/21/31/NA categories) ^d^	4372/141/351/31/4/11	236/20/13/2/1/1	0.0015
Beneficiary Dual Status Code (# of members in 01/02/03/04/06/08/NA categories) ^e^	21/261/25/59/28/366/4150	1/16/1/3/0/13/239	0.62
Co-morbidity Status			
Has Hypertension %	94.10%	96.00%	0.252
Has Pulmonary circulation disorders %	20.30%	40.30%	7.39 × 10–15
Has Chronic pulmonary disease %	49.60%	69.60%	1.86 × 10–10
Has Diabetes %	48.60%	54.20%	0.082
Has Rheumatic arthritis/collagen vascular diseases %	13.10%	9.90%	0.15
Has Renal failure %	38.00%	57.50%	1.76 × 10–10
Has Liver disease %	11.00%	11.00%	1
Has Psychoses %	12.80%	12.80%	1
Has Depression %	32.50%	33.70%	0.73
Has Obesity %	27.00%	34.10%	0.013
# of non-cardiac co-morbidity (mean #)	8.9	10.4	2.16 × 10–10
Charlson Index Score (mean score)	4.89	5.85	2.88 × 10–07
Other Status			
Distance-to-closest-healthcare-facility (mean distance in miles)	8.53	10.37	7.85 × 10–12
Past 12 months total medical charge (mean in dollar)	23,361.56	32,248.25	0.00016

^a^if more than one admission for a member during the total study window, only count the earliest event

^b^T-test for continuous variable and Chi square; Fisher’s Exact test for categorical

^c^Ethnicity code values: 0 = Unknown; 1 = White; 2 = Black; 3 = Other; 4 = Asian; 5 = Hispanic; 6 = North American Native

^d^Indication of the reason for a beneficiary’s entitlement to Medicare benefits as of a particular date as in the following categorie: 10 = Aged without Disabled, and End Stage Renal Disease (ESRD); 11 = Aged with ESRD; 20 = Disabled without ESRD; 21 = Disabled with ESRD; 31 = ESRD; only NA = Not Available

^e^Identifies the most recent entitlement status of beneficiaries eligible for a program(s) in addition to Medicare (e.g., Medicaid). Check Dual Status Codes here: https://www.resdac.org/cms-data/variables/medicare-medicaid-dual-eligibility-code-january

### Features and outcome

**Fig. 1:**
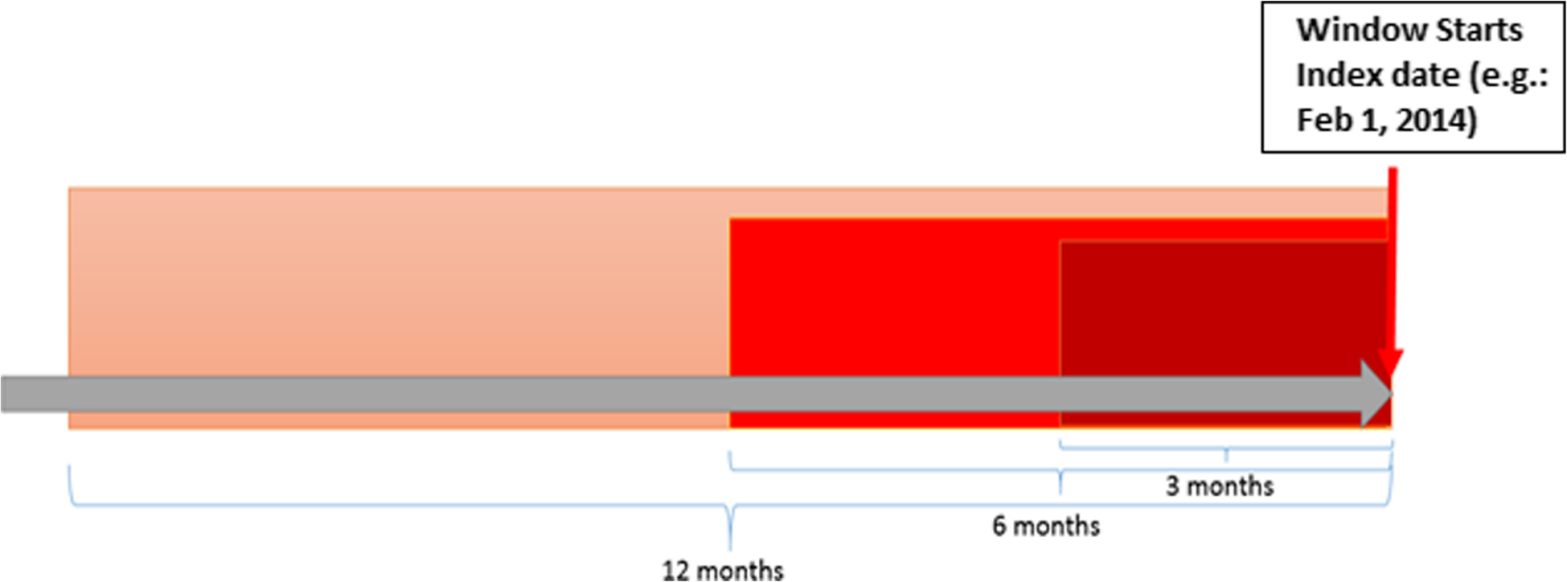
Illustration of feature extraction. Features are extracted at or during the 3 months, 6 months, 12 months, or more prior to the index date. In our Dynamic Random Survival Forest (DRSF) model, the index date is corresponding to the beginning date of each prediction window

We built the prediction model using an auto-extracted candidate feature set. Specifically, seventy-nine features extracted from the CCLF files were classified into several major categories, including demographics, chronic condition, health care service, acute exacerbation record, DME utilization, disease-specific procedure and service, medication, location, and cost [10]. Features were extracted at (for baseline feature, e.g., age, gender and race) or during (for manipulated feature, e.g., count of selected chronic conditions and Charlson Index Score [11]) the 1 month, 3 months, 6 months, 12 months, or 36 months prior to the index date (e.g., February 1, 2014, see Fig. 1) to balance between the comprehensive coverage and the timeliness of the information contained in the feature (Table 2). For example, the historical total expenditure was considered as proxies for overall health condition of beneficiaries and previous health care efficiency; therefore, we collected the total expenditure of beneficiaries during the 12 months prior to the index date for more comprehensive coverage. On the contrary, we preferred more timely information contained in the features such as the most recent care location, medication history, and acute exacerbation record of beneficiaries. Therefore, we collected the features during the 1, 3, or 6 months prior to the index date respectively. The window length selection strategy (as shown in Table 2) could be adjusted by other researchers upon their own discretion of weighing more on timeliness or on comprehensiveness of the information contained in a feature.

**Table 2:** Features pool used in predictive modeling

Feature Category	Features
Demographics	Age, Gender, Race.
Socioeconomics	Medicare status code, Beneficiary Dual Status code
Chronic condition^a^	Any selected chronic conditions^1^; Count of selected chronic conditions^1^; Charlson Index Score [11]
Health care service^b^	Count of a specific health care service utilization, including ED visit^3^, inpatient admission^3^, SNF stay, HHA stay and outpatient physician visit.
Acute exacerbation record^b^	Count of ED visit or inpatient admission with selected exacerbation conditions^2^.
DME utilization^b^	Any DME usage; any oxygen-related DME usage.
Disease-specific procedure and service^c^	Any cardio echo test; any spirometry test; any general pulmonary function test.
Medication^d^	Count of unique prescription.
Location^e^	Most recent care location prior to admission, including home, HHA, SNF, Inpatient and Outpatient
Cost^c^	Total Expenditure

^1^See Additional file 1: Table S1 for chronic conditions used in CHF predictive models

^2^See Additional file 1: Table S2 for exacerbation conditions used in CHF predictive models

^3^We included both the all-cause and disease-specific ED visit/inpatient admission

^a^Such features were collected during the 36 months window prior to the index date

^b^Such features were collected during the 6 months window prior to the index date

^c^Such features were collected during the 12 months window prior to the index date

^d^Such features were collected during the 3 months window prior to the index date

^e^Such features were collected during the 1 month window prior to the index date

Our outcome was defined as the hospital admission with the principal diagnosis code related to CHF. The time to the event of interest was recorded within half a year (6 months or 180 days) from the index date so we had a 6-month-long prediction window. If hospital admission did not happen within the period, it was considered as right-censored in our survival analysis. We did not consider readmission as our outcome of interest, i.e., the admission within 30-days of discharge of the most recent admission. For 30-days readmission events, only the earlier admission was kept as a valid sample and readmissions were dropped. For all other multiple-admissions scenarios, we treated the first admission as a valid sample for predictive modeling.

### Data stream models

Since our data were periodically updated, we adopted the strategy used in the data stream models. The data stream models are developed for the type of data that arrive in a stream or streams. They have been used in areas including sensor data, image data, internet, or web traffic. One common strategy to process such data is to use the sliding window technique [12]. The assumption of imposing sliding windows is that the recent past is more informative for prediction purposes than the older past [12]. The CCLF claim data contain a large number of participants and features, which usually occupy a large amount of computational memory. In addition, the claims data continuously arrive on a monthly basis, which share a similar pattern of data streams. By weighting more on more recent data utilizing the sliding-window technique, we are able to decrease the memory usage, the processing time, and the number of assessment per data window. In brief, we can extract the most important and relevant information from such data in an incremental mode of learning and model adaption.

### Random survival forests

**Fig. 2:**
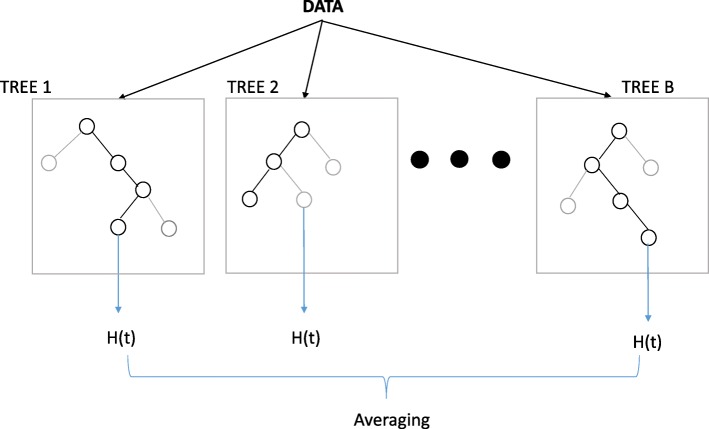
Illustration of Random Survival Forest (RSF) model. Features and Samples are selected by random for each tree. Log-rank splitting is used to grow the tree. At the end of each branch, a cumulative hazard function is calculated for the selected individuals. Finally, the ensembled estimated cumulative hazard function is calculated by averaging over all the trees

RSF method utilizes ensemble trees to analyze the right censored survival data [13]. RSF is closely related to random forests [14] and it inherits many of the good properties from random forests. For example, it is an assumption-free model, which is more flexible than the parametric and semi-parametric survival models, such as Weibull model and Cox proportional-hazards model. It usually performs well when there is a highly non-linear or complex relationship between the features and the response. Moreover, the RSF model incorporates ensemble learning so that it could improve prediction performances from base learners. The mean of cumulative hazard function H(t) is averaged among all the trees for each individual (Fig. 2). It gets the low prediction error from the random draw of the bootstrap sample and the random selection of predictors. In addition, the RSF model uses log-rank splitting developed from a well-accepted non-parametric log-rank test. In this paper, we used an R package randomForestSRC (version 2.2.0 https://cran.r-project.org/web/packages/randomForestSRC/index.html) to fit the RSF model. It is open-source and freely available from the Comprehensive R Archive Network (CRAN).

### Dynamic random survival forests model

**Fig. 3:**
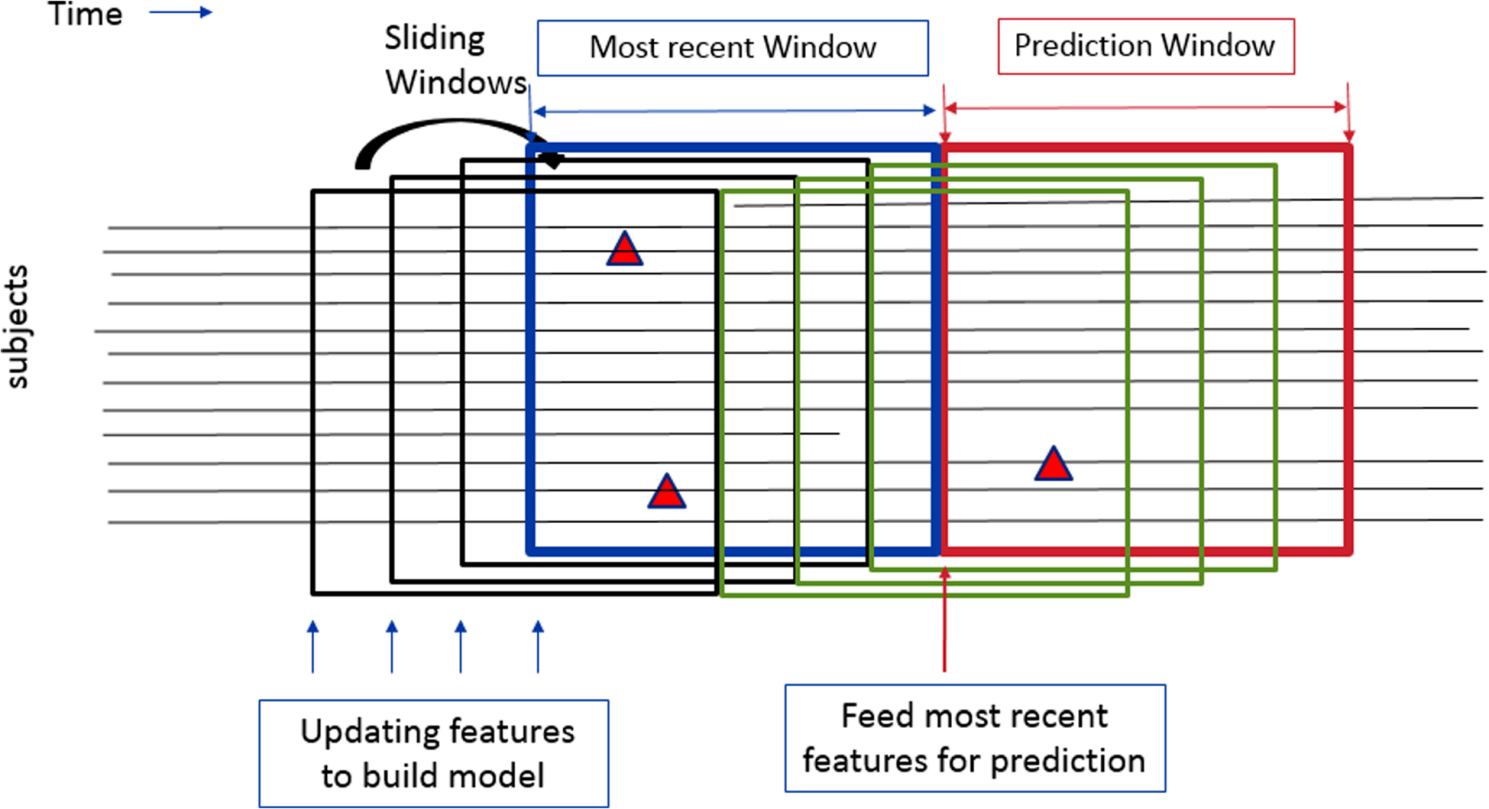
Demonstration of the Dynamic Random Survival Forest (DRSF) Model with sliding windows. The black lines represent different subjects at risk. The red triangles represent the onset of adverse event, e.g. hospital admission. The red box represents the prediction window of interest, while the blue box represents the prediction window for model training and validation purpose corresponding to the red box. At the beginning of the red and blue windows, i.e., the index dates, features are auto-extracted for the model building and prediction (see section “Features and Outcome”). The black and the green boxes are the historical sliding windows trained and predicted following the same concepts as the blue and the red box except they were earlier on the time axis. The green boxes and red box are further combined for the ensemble estimation of the hazard function and survival function at different time points within the red box

The DRSF model carries the advantage of properties of RSF model and sliding window technique. We built the DRSF as in the following steps (Fig. 3): first, we extracted features prior to the index date where the prediction window begins as discussed above (also shown in Table 2 and Fig. 1); second, we trained and validated the model for the specific window using RSF. The variable selection and model building procedure was described in detail later; third, we predicted the survival rate and calculated the prediction accuracy in the following testing window (no overlap with the previous window used for model training and validation); fourth, we moved forward the window by one month and repeated the process until the end of the overall study time; finally, we obtained the prediction score from each single window and combined the information of nearby windows (ensemble windows) to build a classification model. More specifically, for each individual at time *t*, let *Wτ* denotes the set of all time points: *W*_*τ*_ = { *W*_*t*_ |*τ ϵ* [*t*, *t* + *∆t*] }. The hazard rate for individual *i* at time *t* can be calculated by the combination of different nearby windows: $h(t)=\frac{1}{N}{\sum}_{Wt\in W\tau}h(t)$, N denotes the total number of windows available for each individual in time *W*_*τ*_.Thus, we can get the survival function as *S*(*t*) = exp(− ∑ *h*(*t*)). The combination procedure allows that more recent window have higher weights in calculating the hazard rates and the survival function.

### Model building and selection

We built our model by a two-step feature selection method using features in the previously defined feature pool. In the first step, features with variable importance less than 0 were excluded. In the second step, we performed a nested sequence of models starting with the top variables, e.g., with top two variables, three variables and so on [13]. We finalized our feature selection process by choosing the features until there was little or no incremental effect of the features. Each window was allowed to have different features.

### Model evaluation

We adopted two different statistics to compare among the methods in benchmark. Firstly, we included the concordance summary C-statistics, which is directly related to Kendall’s tau. It can be interpreted as the probability that the marker value for a randomly selected case exceeds the marker value for a randomly selected control. The marker value for cox-proportional hazard is the cox linear predictor (xb), the marker value for penalized linear regression (i.e., batch-mode L1-penalized logistic regression) is the predicted value (i.e., the logarithm odds), and the marker value for RSF is the ensemble mortality, proposed by Ishwaran et al. and defined as the expected value for the cumulative hazard function [13, 15]. Secondly, we included the cumulative/dynamic time-dependent AUC evaluated at 60 days and 180 days. The concordance summary C-statistics is a weighted integral function of AUC [15]. The cumulative/dynamic time-dependent AUC cannot be directly compared to logistic regression, while the C-statistics is comparable. More details can be found in Additional file 1: S1 Evaluation Methods.

We used 67% samples of one prediction window to train and the rest 33% samples, the out-of-bag data, to validate for RSF. Penalized cox-regression and logistic regression were trained and validated using the standard cross-validation procedure embedded in the R package Glmnet. (available at https://cran.r-project.org/web/packages/glmnet/index.html) The model was further tested for their prediction error in the contiguous prediction window. For example, for the first window, the training and validating window began with the index date of Feb 1, 2014, we collected features before this date and collected events as endpoint after this date until Jul 31, 2014, to train and validate the RSF model (supervised learning). Then, the contiguous prediction window started from the index date of Aug 1, 2014 and ended on Jan 31, 2015. Again, we collected features before this index date and updated the previously trained model by these features to obtain the predicted events. We compared the predicted events with the true events happened between Aug 1, 2014 and Jan 31, 2015 and calculated the prediction error as the testing error.

Besides using the original dataset in model benchmark, we adopted a bootstrap strategy to obtain the confidence interval of the performance score. Specifically, we used the bootstrap procedure to calculate the confidence interval of the C-statistics and AUC based on 500 bootstrap samples. The steps are described as follows: 1), Fit the model on the training dataset; 2), Randomly select case and controls separately with the number equal to that in the testing dataset, i.e., stratified sampling with replacement; 3), Calculate the marker value for DRSF and competing methods; 4), Calculate the AUC and C-statistics for each bootstrapped dataset, then extract the 2.5% and 97.5% of the statistics as the confidence interval.

## Results

### Dynamic random survival Forest model with Most recent window

When the number of windows in the DRSF model reduces to one, it is equivalent to the regular RSF model with the features extracted from the most recent window (as in Fig. 3). It relies on the assumption that the latest information provides enough prediction power of the event and the historical data cannot further improve the prediction power. We presented the results of multiple DRSF models with the most recent window across the timeline as in Table 3. From Feb 1, 2014 to Sep 1, 2014 as the index date of the window, we were able to collect 8 such individual models. The Harrell’s C statistics of such models ranged from 0.66 to 0.71. The AUC at the 60th day and the AUC at the 180th day indicate that longer prediction was not more accurate than shorter prediction or vice versa. However, we observed more stable estimates of AUC at the 180th day with a narrower 95% confidence interval than those AUC estimates evaluated at the 60th day. One of the reasons could be that more events happened up to the 180th day than up to the 60th day, which increased the outcome frequency and thus reduced the prediction variance.

**Table 3:** AUC and C-statistics for DRSF model with the most recent window

Training /Testing Window Index Date	Evaluation window Index Date	# of subjects in evaluation	# of events in evaluation	Mean time to event	AUC at the 60th Day	AUC at the 180th Day	Harrell’s C statistics	# of covariates^1^
Feb 1,14	Aug 1,14	5175	263	175.17	0.67^a^ (0.61,0.73)^b^	0.64 (0.60,0.67)	0.66 (0.63,0.70)	14
Mar 1,14	Sep 1,14	5143	250	175.14	0.67 (0.61,0.72)	0.67 (0.64,0.71)	0.68 (0.65,0.72)	17
Apr 1,14	Oct 1,14	5069	247	175.07	0.70 (0.65,0.75)	0.68 (0.65,0.71)	0.68 (0.65,0.71)	14
May 1,14	Nov 1,14	4988	230	175.31	0.65 (0.60,0.71)	0.66 (0.63,0.70)	0.69 (0.66,0.72)	20
Jun 1,14	Dec 1,14	4958	225	175.30	0.66 (0.61,0.71)	0.65 (0.62,0.70)	0.69 (0.65,0.72)	24
Jul 1,14	Jan 1,15	4261	201	175.20	0.74 (0.68,0.79)	0.69 (0.66,0.73)	0.71 (0.67,0.74)	16
Aug 1,14	Feb 1,15	4233	176	175.70	0.71 (0.65,0.77)	0.69 (0.65,0.72)	0.71 (0.67,0.74)	24
Sep 1,14	Mar 1,15	4222	172	175.85	0.61 (0.55,0.67)	0.65 (0.61,0.69)	0.68 (0.64,0.71)	14

A larger AUC or C statistics represents a better model prediction performance

^1^Number of covariates selected by RSF model

^a^Score obtained with the original dataset

^b^95% confidence interval of the score obtained with 500 bootstrapped datasets

### Comparison of the models

**Fig. 4:**
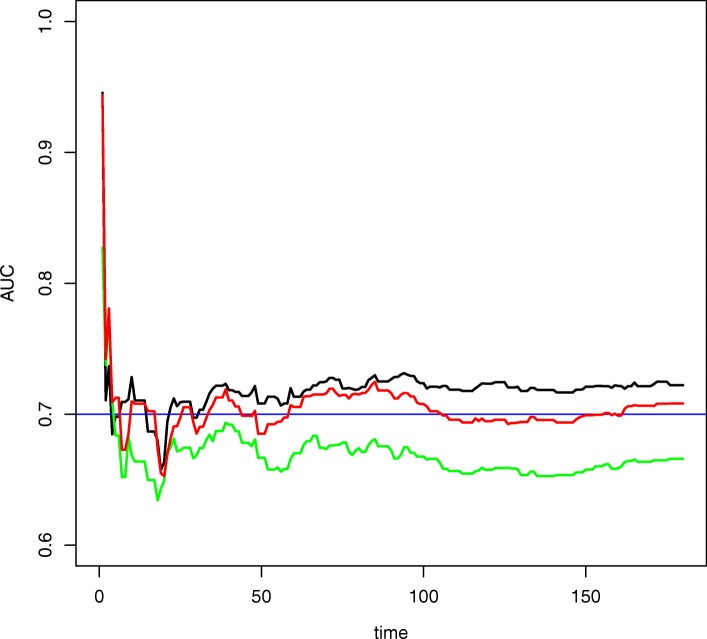
Comparison of the prediction power among DRSF model with 5 windows, Penalized Cox regression model with most recent window (1 window), and the batch-mode RSF model using the testing window from March 1st, 2015 to August 31st, 2015. The black line is the AUC curve of DRSF with 5 windows, the red line is the AUC curve of Penalized Cox regression with 1 window, and the green line is the AUC curve of batch-mode RSF model. The x-axis represents the number of days since March 1st, 2015. The y-axis represents the Area-Under-the-ROC-Curve (AUC)

We further accessed the performance of the DRSF model with different numbers of windows and compared it with the benchmark models as in Fig. 4 and Table 4. The testing dataset (window) for all models were the same and recorded patient events from Mar 1st, 2015 to Aug 31st, 2015. In Fig. 4, The first benchmark model (AUC curve represented by a green line) was the batch-mode RSF model using baseline features collected prior to Feb 1st, 2014 and all historical outcome data from Feb 1st, 2014 to Feb 28th, 2015 for training and validation purpose, which has been commonly used in applied survival literature [16, 17]. The second benchmark model (AUC curve represented by a red line) was the penalized Cox regression with (most recent) 1 window. In a Cox model, the unique effect of a unit increase in a covariate is multiplicative with respect to the hazard rate. Additionally, L1-norm type constraint could be added to the Cox partial log-likelihood to enable model selection [18]. In short, Fig. 4 showed that the time-dependent AUC of DRSF with 5 windows (AUC curve represented by a black line) was consistently larger than both the batch-mode RSF model and the penalized Cox regression with the most recent window (1 window). It suggested that the utilization of time-varying features via ensemble windows helped improve the model performance. Table 4 illustrated the numerical results including AUC at the 60th day, AUC at the 180th day and Harrell’s C statistics, by comparing five different DRSF models with an increasing number of ensemble windows and four benchmark models. We have adopted the same testing window, which covered days from March 1st, 2015 to August 31st, 2015, for all benchmark models to measure performance. Four benchmark models included the batch-mode RSF model using baseline features (also in Fig. 4), the penalized Cox regression with the most recent window (also in Fig. 4), the batch-mode penalized Cox regression using baseline features and the penalized logistic regression with the most recent window. Table 4 showed that both the point AUC and C-statistics of the DRSF models with two to five windows were larger than those of the batch-mode RSF model. For example, comparing to the batch-mode RSF model, the C-statistics of DRSF with 5 windows increased by 6% (from 0.67 to 0.71) and the AUC at 60th day increased by 6% as well (from 0.67 to 0.71). Besides, we did observe an increasing trend of performance as the number of windows kept increasing for DRSF models. A breakdown analysis of these involved sliding windows as in Additional file 1: Figure S1 revealed that the 1st (most recent) sliding window actually had a relatively weak performance of prediction regardless of selected models, which was validated in Table 4 that DRSF with 1 window model rendered bottom performance. Additional file 1: Figure S1 also revealed that from the 2nd sliding window to the 6th sliding window, all windows have relatively high performance of prediction. As a validation, we could see DRSF with 2 windows has an obvious performance boost over DRSF with 1 window in Table 4. The contingent 5 windows with high prediction performance also helped explain the observed increasing trend of performance as the number of windows kept increasing for DRSF models (i.e., from DRSF with 1 window to DRSF with 5 windows). The windows that have longer distance to the testing window, such as the window of 2014-03-01 (the 7th) and the window of 2014-02-01 (the 8th), have relatively low performance. We speculated the low performance was due to the fact that the earlier information contained in the earlier window will not add value to the prediction or classification task of future events, as signal diluted out and noise dominated in earlier windows. On the contrary, the low performance of the 1st (most recent) window may be due to randomness, e.g., sparser outcome in the window coverage or sparser predictor information collected before the index date of the window. We thus inferred the performance of DRSF model may depend on the tuning of both the window size and the number of ensemble sliding windows. For other types of benchmark models, the penalized logistic regression (PLS) with the most recent window (1 window) operated similarly as the DRSF with 1 window method in feature collection but differs in the way of prediction. Specifically, DRSF with 1 window method predicts the event at all time points within the prediction window and has taken into consideration the time-to-event information, as most survival analysis related method could do; on the contrary, PLS with 1 window method predicts binary event could only provide point estimate at requested time points. Since the PLS cannot provide an overall estimation on all time points, it requires model fitting for each time point of interest within the prediction window, thus computationally inefficient. Multiple fitting of penalized logistic regression could also lead to inconsistent conclusions among multiple predictions at different time points. On the other hand, DRSF model with five windows has comparable performance to the penalized logistic regression and is immune to these drawbacks. Two other benchmark models were penalized Cox models, i.e., the batch-mode Cox model and the Cox with 1 window model. While both Cox models have high performances, especially the batch-mode Cox model has 0.72 as the score for all three metrics, which nearly equates the performance of DRSF with 5 windows model, we concerned about the potential violation of the proportional hazard assumption when generalizing this method to other applications in real-world datasets. Due to the (semi) parametric nature of the Cox model, if the model assumption by large holds, the penalized Cox model may have a better performance than the RSF model, which is nonparametric and has traded the prediction power for model robustness. We certainly can incorporate Cox model with the sliding window technique and may observe a higher performance, like what we did with RSF and see performance increased on DRSF models. However, for sliding window concept demonstration and in favor of a more robust model (with fewer model assumptions), we presented the work of DRSF with multiple sliding windows. In summary, the results showed that our DRSF model with 5 windows had an overall better performance than the other benchmark models. Given the robustness of DRSF models, we prefer such methods even when parametric models could yield a similar performance. We also inferred that a finer tuning of both the window size and the number of ensemble sliding windows could yield a better optimized DRSF model.

**Table 4:** AUC and C-statistics for DRSF models with different number of ensemble windows and benchmark models on the testing window covering days from March 1st, 2015 to August 31st, 2015

Models	AUC at the 60th day	AUC at the 180th day	Harrell’s C statistics
Batch-mode RSF	0.67^a^ (0.61,0.72)^b^	0.66 (0.63,0.70)	0.67 (0.63,0.71)
Batch-mode Cox^1^	0.72 (0.65,0.76)	0.72 (0.69,0.76)	0.72 (0.69,0.76)
Cox^1^ with 1 window	0.63 (0.63,0.74)	0.70 (0.66,0.74)	0.71 (0.67,0.74)
PLS with 1 window^2^	NA	NA	0.71 (0.67,0.74)
DRSF with 1 window	0.61 (0.55,0.67)	0.65 (0.61,0.69)	0.68 (0.64,0.71)
DRSF with 2 windows	0.67 (0.62,0.71)	0.68 (0.65,0.71)	0.70 (0.67,0.72)
DRSF with 3 windows	0.69 (0.66,0.72)	0.68 (0.63,0.72)	0.70 (0.67,0.73)
DRSF with 4 windows	0.67 (0.63,0.72)	0.7 (0.67,0.73)	0.70 (0.67,0.73)
DRSF with 5 windows	0.71 (0.66,0.75)	0.71 (0.68,0.74)	0.71 (0.68,0.74)

Note: A larger AUC or C-statistics represents a better model prediction performance

^1^Cox: Here we used penalized Cox proportional hazard model

^2^PLS with 1 window: Cumulative time-dependent AUC at a specific time point is not available in penalized logistic regression model. Thus, we put NA as Not Available here

^a^Score obtained with the original dataset

^b^95% confidence interval of the score obtained with 500 bootstrapped datasets

### Variable importance

**Fig. 5:**
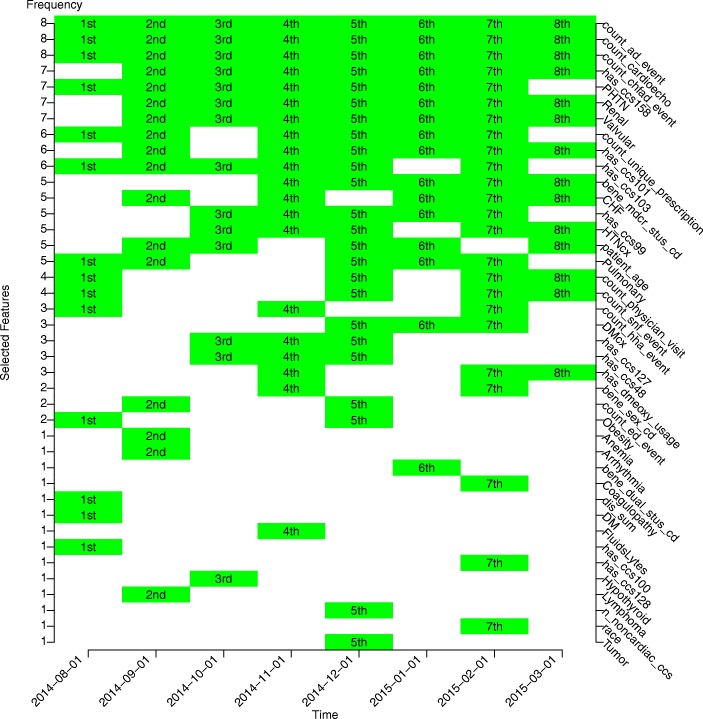
Variables selected in different testing windows with the index date ranging from August 1st, 2014 to March 1st, 2015 by the increment of a month. Highlighted block indicates a specific variable (variable name annotated along the rightmost y-axis) was selected at a specific window (window index date annotated along the x-axis) by the DRSF model with 3 windows. The leftmost y-axis represents the frequency a variable was chosen among eight available windows. The description of each variable is listed in the Additional file 1: Table S3

To further show the validity of the model, we presented the variables selected in the testing data sets at multiple windows in Fig. 5, demonstrating that different windows could share a common set of features and meanwhile own their unique features. Here, we defined two measures of variable importance based on the DRSF model. Local variable importance, defined as the importance of a variable in a single window (equivalently in a single time period), can be evaluated by the number of times a variable is selected from multiple ensemble trees in that single window. For example, a variable chosen 90% of the 500 trees is usually considered more important than a variable chosen 10% of the 500 trees. This practice is commonly applied with random forest related methods. On the other hand, we define the global variable importance as the importance of a variable across windows (equivalently across times), which can be evaluated by the number of windows where the variable is selected. For example, in this study, if we set the selection criteria of a variable from a single window is at least 30% (arbitrarily defined by the user) out of the 500 trees and we saw such a variable was selected 6 out of 8 windows (given at each window greater than 30% tree vote), we could infer this variable is important about predicting the endpoint most of the time in the total time span covered in this study. This practice is more novel due to the sliding-window nature of our method, and more valuable in evaluating whether a variable is all-time important or “seasonal” important. For instance, previous utilization such as cardio echo tests and acute inpatient visits were selected through all the windows, suggesting that the two features were essentially informative regarding prediction of future CHF inpatient onset all the time. Similarly, a few prior diagnoses of chronic conditions and evidence of comorbidities, such as chronic kidney disease, pulmonary circulation disorders, renal failure, and valvular disease, are also predictive of future inpatient onset most of the time (7 out of 8 windows). Furthermore, taking more types of prescription drugs, which may reflect the severity of the health condition, was also associated with future inpatient onset most of the time (6 out of 8 windows). On the other hand, tumor history, only selected at the 5th window, could be a trivial signal. With more windows assessed, we could have a more comprehensive assessment of variable importance.

### Dynamic monitoring of the risk of hospitalization

**Fig. 6:**
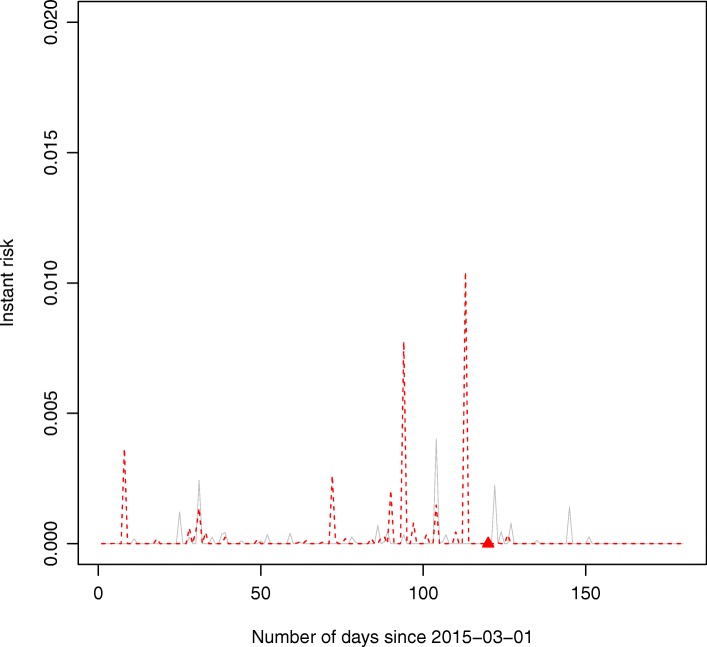
Dynamic monitoring the risk of hospitalization due to congestive health failure. The red dash line represents the instant risk (ensembled hazard rate) of a randomly selected subject who had an admission event at the 120th days in reality (marked as the red triangle on the horizontal line); the grey solid line represents the instant risk of another randomly selected subject who did not have such an event onset during the prediction window in reality

Figure 6 shows the risk stratification for two randomly chosen subjects based on their (ensembled) hazard function at each time point within the prediction window time period. The individual-level instant risk is also valuable for dynamic monitoring of the risk of hospitalization for beneficiaries. Figure 6 exhibits the continuous risk curve from one randomly selected beneficiary who had hospitalization during the time window (red) and another beneficiary who did not have such event occurred (grey) during the time window. It demonstrated that the subject got hospitalization within the time window had overall higher continuous risk curve than the other randomly selected subject who did not have hospitalization. Additionally, the high peak of the red curve almost coincides with the true event (red solid triangle mark) onset time (the 120th day since Mar 1st, 2015), which shows the effectiveness of using DRSF model to monitor the health condition of beneficiaries.

## Discussion

Clinical practice and research are now faced with the increasing challenges of processing complex data. Machine learning technique applied to medical data is a recent area of research that aims to provide better knowledge extraction and evidence-based clinical decision support. In this paper, we proposed a novel DRSF model for risk prediction and classification by taking full advantage of the monthly-updated claim data. We showed that it had a clear edge over the existing methods. First, our method can provide risk classification for every time point in the defined time intervals and it does not require multiple model fitting, which makes the computation efficient and the interpretation consistent. Models without taking into account the time-to-event information, such as commonly used PLS, lack such property [16, 19, 20]. Secondly, our model has fewer assumptions than the parametric or semi-parametric models, such as Cox proportional hazard models, which make it robust to the inherent complicated nature of the healthcare data. Our method enables us to select features automatically from a large feature pool without concerning much model assumption violation, yet it could provide good prediction power. Thirdly, our model can use time-varying and time-sensitive features through sliding-window and window ensemble approaches to improve prediction power, as compared to the static RSF model built on all historical endpoints and the baseline features. As shown in Table 4, updating features with time by ensembling windows could help build a predictive model with a higher predictive power. Our model is able to utilize both the historical data and most recent data to achieve the optimal prediction power. We used window sizes as 6 months and the number of windows of five to demonstrate the model performance of the DRSF. Finer tuning of the two parameters may yield a better optimized DRSF model. We emphasize the novelty of combining sliding window with the survival model to deal with large-scale data. We mainly focused on RSF due to its robustness in model building, but we also showed the potential of combining sliding window with penalized Cox model.

Our DRSF model yielded clinically interpretable results. In general, we classified all features into ten categories as reported in Table 2. Figure 5 revealed that the “Health care service”, “Disease-specific procedure and service”, “Chronic conditions”, “Medication” and “Socioeconomics” are the most contributing feature categories across different testing windows. For example, “Health care service” included the count of past admissions (count_ad_event) and the count of past CHF-related admissions (count_chfad_event) as the most contributing features, we interpreted them as acute care utilization history and could reflect a beneficiary’s future utilization need; “Disease-specific procedure and service” included the count of cardio echo tests (count_cardioecho) as the most contributing feature, which is a typical test for beneficiaries with CHF comorbidity; “Chronic conditions” included the Chronic Kidney Disease (has_ccs158), Pulmonary Circulation Disorders (PHTN), etc., which were well-known comorbidities accompanying CHF and increased the clinical complexity of the beneficiary; “Medication” included the count of unique prescriptions given to a beneficiary (count_unique_prescription), which could reflect the disease status complexity of a beneficiary; “Socioeconomics” included beneficiary’s Medicare status code (bene_mdcr_stus_cd), which was typically used as a proxy to reflect beneficiary’s socioeconomics and functional status. These findings confirmed the DRSF models’ effectiveness and provided insights into the mechanism of hospitalization.

We are aware of the limitations of our current model. First of all, our model did not take into account multiple admissions (after excluding the 30-day readmission) per person within the specified time period, i.e., recurrent events. In our study, the average admission rate is around 5% in all time windows and there are very few beneficiaries with more than one admission. However, for other studies with a noticeable portion of recurrent events existing in the time windows, our method could still be useful by adjusting the window size or using the parametric recurrent event model. Therefore, Beneficiaries with more than one admission can be considered as having higher risks than those with only one admission or with no admission. Secondly, we could obtain the local and global variable importance but not the effect size measure for each variable. In the study, our major interest is to predict the risk for beneficiaries and monitoring their health conditions. However, if the research purpose is to quantify the feature effect, our model could not provide the effect size measure because the model is distribution free. Instead, we can extend our methodology to parametric or semi-parametric survival model, which gives the quantification of effect size measure and embraces even faster computing speed. However, it has to be used with caution because the model assumptions for parametric models are less likely to meet when a large number of parameters are involved. Thirdly, we did not have enough data (claim coverage of more months) for fine-tuning the window size or number of windows used for ensemble combination to achieve a better performance of the model. For example, fine-tuning of these parameters could extract the most relevant historical medical information for prediction purpose. If the data is too historical to contribute valuable signal, combining such data in the time window would increase noise and diminish the model performance [21]. On the contrary, skipping useful information in the recent data will also lead to the reduced model performance. In our study, the 2nd to 6th most recent windows contained additional useful information (as in Additional file 1, Figure S1), which improved the performance of the DRSF model with greater than one sliding window as compared to the DRSF model using the most recent window. In the meanwhile, the usefulness of the historical information gradually dimed out and the noise dominated the signal (e.g., the 7th and 8th window). Fourthly, with claim coverage of more months, we could apply data-adaptive weights for different windows rather than taking the average of hazard functions to combine ensemble windows. Lastly, we could consider applying the Bayesian framework to the sliding-window platform. Instead of averaging, we could take into account prior knowledge when analyzing data and turn the data analysis into a process of updating that prior knowledge with biomedical and health-care evidence [22].

## Conclusion

We presented an efficient and flexible model developed for periodically updated data such as the monthly updated claim feed data released by the CMS to predict the risk of hospitalization. By combining sliding window technique and RSF, our model can achieve good prediction power, provide measure of variable importance and real-time monitoring of the health condition of high-risk patients. Instead of using all historical data, we showed that our model only utilizes several most recent windows such that it can save computational memory and power. In the meanwhile, the model can be easily extended to handle other types of outcome of interest such as continuous variable or count type variable. In the long run, we hope the successful prevention of adverse events could help reduce financial burdens of payers and increase the health condition of the targeted population.

## Additional file

Additional file 1: **S1.** Evaluation Methods. **Figure S1.** Confidence intervals of C-statistics for Random Survival Forest models trained from different training windows and tested on the same testing window. **Table S1.** Chronic Conditions used in CHF Predictive Modeling. **Table S2.** Acute Exacerbation Conditions used in CHF Predictive Modeling. **Table S3.** Description of Selected Features (DOCX 60 kb)

## Abbreviations

ACO: Accountable Care Organization
AUC: Area under the ROC curve
CCLF: the Claim and Claim Line Feed
CCS: Clinical classifications software
CHF: Congestive heart failure
CMS: The Centers for Medicare and Medicaid Services
DME: Durable medical equipment
DRSF: Dynamic random survival forests
HHA: Home Health Agency
ICD-9-CM: International Classification of Diseases, Ninth Revision, Clinical Modification
PLS: Penalized logistic regression
ROC: Receiver operating characteristic
RSF: Random survival forests
SNF: Skilled nursing facility
