# Supplementary material for: Dynamic prediction of hospital admission with medical claim data
Source: BMC Med Inform Decis Mak. 2019 Jan 31;19(Suppl 1):18. doi: 10.1186/s12911-019-0734-y (PMC6354329; doi:10.1186/s12911-019-0734-y)
Supplement: Supplementary file 1 — S1. Evaluation Methods. Figure S1. Confidence intervals of C-statistics for Random Survival Forest models trained from different training windows and tested on the same testing window. Table S1. Chronic Conditions used in CHF Predictive Modeling. Table S2. Acute Exacerbation Conditions used in CHF Predictive Modeling. Table S3. Description of Selected Features (DOCX 60 kb) [file 12911_2019_734_MOESM1_ESM.docx]

S1 Evaluation Methods

As suggested by Heagerty [1], we used nearest-neighbor estimator to estimate the ROC curve in order to guarantee that sensitivity and specificity are monotone. In addition, we used cumulative sensitivity and dynamic specificity in order to distinguish subjects failing by a given time and those failing after this time.

Cumulative Sensitivity(c,t) = $P\left( M_{i}(t)>c | T_{i}\leq t \right)$

Dynamic Specificity(c,t) = $P\left( M_{i}(t)\leq c | T_{i}>t \right)$

$M_{i}\left( t \right)$ is the 1-survival function at time t because higher survival function means lower risk. c is the cutoff point for $M_{i}\left( t \right)$.

Since the AUC is a measure of the discrimination ability for each time point, it is a good tool to use if we have mulitple time points of interest. Please note that the definition of AUC of the survival model is different from non-survival models, such as logistic regression or PLS. Therefore, the AUC is not directly comparable between the two types of models.

If all time points are of interest, the concordance summary C statistics, which can be interpreted as the probability that the marker value for a randomly selected case exceeds the marker value for a randomly selected control, can be used to access the overall model performance. This statistics is comparable among different types of models.

More specifically, we used the internal cindex function from R package randomForestSRC to calculate the concordance summary C statistics; we used the survivalROC.C function from R package survivalROC to evaluate the dynamic time-dependent AUC.

Figure S1 **Confidence intervals of C-statistics for Random Survival Forest models trained from different training windows and tested on the same testing window**

X-axis represents the index dates of different training windows; y-axis represents the C-statistics for RSF models trained from different training windows and tested on the same testing window with the index date of 2015-03-01. Each bar of C-statistics represents 95% confidence interval from 500 bootstrapped datasets.


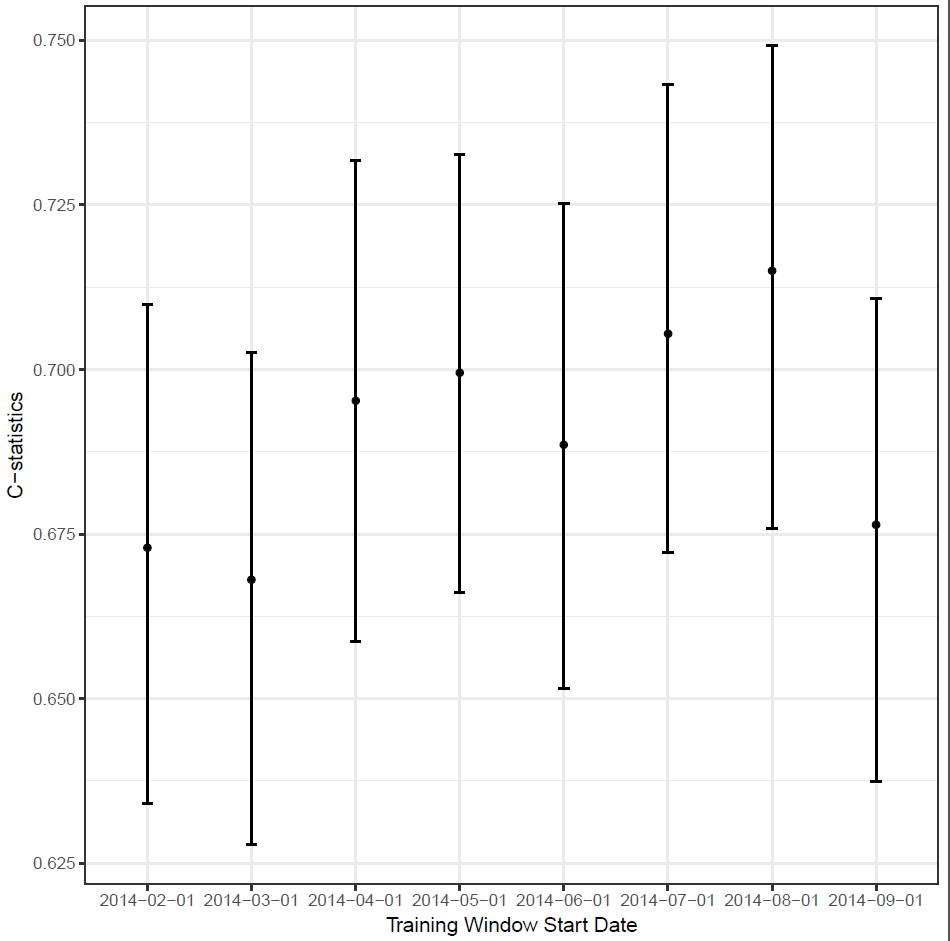


Table S1 **Chronic Conditions used in CHF Predictive Modeling**

| **CCS^1^ ID** | **CCS Description** |
| --- | --- |
| **48** | Thyroid Disorder |
| **49** | DiabMel no c |
| **50** | DiabMel w/cm |
| **53** | Hyperlipidem |
| **59** | Anemia |
| **79** | Parkinson-s |
| **87** | Retinal dx |
| **88** | Glaucoma |
| **98** | HTN |
| **99** | Htn complicn |
| **100** | Acute MI |
| **101** | Coron athero |
| **102** | Chest pain |
| **103** | Pulm heart dx |
| **111** | Other CVD |
| **114** | Perip athero |
| **127** | COPD |
| **128** | Asthma |
| **158** | Chr kidney disease |
| **205** | Back problem |
| **206** | Osteoporosis |
| **227** | Spin cor inj |
| **651** | Anxiety disorders |
| **653** | Delirium/dementia/amnestic/other cognitiv |

^1^ The Clinical Classifications Software (CCS) for ICD-9-CM was developed and is maintained by the Healthcare Cost and Utilization Project (HCUP), which is sponsored by the Agency for Healthcare Research and Quality (AHRQ).

Table S2 **Acute Exacerbation Conditions used in CHF Predictive Modeling**

| **CCS ID** | **CCS Description** |
| --- | --- |
| **2** | Septicemia (except in labor) |
| **129** | Aspiration pneumonitis; food/vomitus |

Table S3 **Description of Selected** **Features**

| **Feature** | **Description** |
| --- | --- |
| **count_ad_event** | Count of previous general admission events |
| **count_cardioecho** | Count of previous cardio echo tests |
| **count_chfad_event** | Count of previous CHF-caused admission events |
| **has_ccs158** | Has prior diagnosis of “Chronic kidney disease” |
| **PHTN** | Has comorbidity history of “Pulmonary circulation disorders” |
| **Renal** | Renal failure |
| **Valvular** | Valvular disease |
| **count_unique_prescription** | Count of previous unique prescriptions |
| **has_ccs101** | Coronary atherosclerosis |
| **has_ccs103** | Pulmonary heart disease |
| **bene_mdcr_stus_cd** | Beneficiary’s Medicare eligibility status code. Usually it reflects a member’s socioeconomic status and disability status |
| **CHF** | Congestive heart failure |
| **has_ccs99** | Hypertension with complications and secondary hypertension |
| **HTNcx** | Hypertension, complicated |
| **patient_age** | Age of patient at the time of study |
| **Pulmonary** | Chronic pulmonary disease |
| **count_physician_visit** | Count of previous outpatient physician visits |
| **count_snf** | Count of previous Skilled Nursing Facility visits |
| **count_hha_event** | Count of previous HHA visits |
| **DMcx** | Diabetes, complicated |
| **has_ccs127** | Chronic obstructive pulmonary disease and bronchiectasis |
| **has_ccs48** | Thyroid disorders |
| **has_dmeoxy_usage** | Has any oxygen-related DME usage |
| **bene_sex_cd** | Gender |
| **count_ed_event** | Count of previous emergency department visits |
| **Obesity** | Obesity |
| **Anemia** | Deficiency anemias |
| **Arrhythmia** | Arrhythmia |
| **bene_dual_stus_cd** | Beneficiary’s Medicare and Medicaid dual eligibility status code. Usually it reflects a member’s socioeconomic status and disability status |
| **Coagulopathy** | Coagulopathy |
| **Dis_sum** | Average distance (in miles) to close by health care facilities |
| **DM** | Diabetes, uncomplicated |
| **FluidsLytes** | Fluid and electrolye disorders |
| **has_ccs100** | Acute myocardial infarction |
| **has_ccs128** | Has Asthma as chronic condition |
| **Hypothyroid** | Hypothyroidism |
| **Lymphoma** | Lymphoma |
| **n_noncardiac_ccs** | Number of non-cardiac CCS conditions a member has |
| **race** | ethnicity |
| **Tumor** | Solid tumor without metastasis |

**Reference**

1. Heagerty PJ, Zheng Y. Survival Model Predictive Accuracy and ROC Curves. Biometrics. 2nd ed. Blackwell Publishing; 2005 Mar;61(1):92–105.
